# The impact of COVID-19: variations in volumes and characteristics of retina surgeries

**DOI:** 10.1186/s12893-022-01499-x

**Published:** 2022-02-05

**Authors:** Julie M. Shabto, Christian Faaborg-Andersen, Ghazala A. O’Keefe

**Affiliations:** 1 Emory University School of Medicine, Atlanta, GA USA; 2 Department of Ophthalmology, Section of Vitreoretinal Surgery and Diseases, Section of Uveitis and Vasculitis, Emory University School of Medicine, Atlanta, GA USA

**Keywords:** COVID-19, Standards, Retina, Surgery, Time-to-treatment

## Abstract

**Background:**

In concordance with medical recommendations in response to COVID-19, Emory Healthcare limited surgical procedures starting March 16, 2020. We investigated the impact of these recommendations on the number, types, and urgency of surgical retina cases.

**Methods:**

We conducted a retrospective review of all surgical patients at the Retina division of the Emory Eye Center from February 17–April 12, 2020 and during the same time period in 2019 and 2018. The demographics of patients and the number, types and urgency of retina surgeries were collected. Descriptive statistics for each variable were reported. Univariate analysis was carried out using the chi-square test or Fisher’s exact test for categorical covariates.

**Results:**

From February 17–March 15 to March 16–April 12, 2020, total surgeries decreased from 87 to 34. Emergent cases, occurring within 7 days of surgical order placement, decreased from 23 to 18 (p = 0.0056), and urgent cases, occurring within 21 days of surgical order placement, decreased from 26 to 4 (p = 0.0380). From March 16–April 12, 2019 there were 62 surgeries: 21 emergent (34%), 14 urgent (23%). From March 16–April 12, 2018 there were 68 surgeries: 15 emergent (22%), 21 urgent (30%). After March 16, 2020, average patient age decreased from 39.4 to 25.7 years. There were no statistically significant differences in racial make-up or insurance coverage for those having surgery prior to versus after March 16, 2020.

**Conclusion:**

National recommendations for ophthalmologic surgeries during COVID-19 disproportionately affected older patients and patients with urgent cases at our tertiary care academic medical center. These results may inform the ophthalmologic field of the potential effects of pandemics such as COVID-19 on the surgical retina care of patients.

## Introduction

In response to the ongoing COVID-19 pandemic, ophthalmologists across the United States had to cancel surgical procedures, modify schedules, and determine which patients necessitated clinic visits and which did not. The American Academy of Ophthalmology (AAO) and the Centers for Disease Control (CDC) recommended limiting procedures with the potential for aerosolization of viral respiratory droplets, including any procedure requiring anesthesia. On March 18, 2020, the AAO and the American Society of Retina Specialists (ASRS) made official recommendations for ophthalmologists to cease treatment deemed non-urgent or elective [1, 2]. Emory Healthcare limited patient visits and surgical procedures starting March 16, 2020 in concordance with national medical recommendations.

The definition of non-urgent or elective varies widely depending on the specialty. Patients frequently receive general ophthalmic care from non-eye care providers, including in the Emergency Department (ED). Frequently, the designation of non-urgent versus urgent cannot be made before an examination due to vague presenting complaints. While cancelling non-urgent and elective surgical procedures helped reallocate resources for COVID-19 patients, urgent and emergent care needs may continue at their previous rates or increase either immediately or soon after the lockdown is lifted.

In order to assess the effect of medical recommendations in response to COVID-19 on surgical retina procedures, we compared the volume, type, and urgency of surgical procedures at our academic medical center prior to and during the COVID-19 shelter-in-place in Atlanta, GA, starting March 16, 2020. We also compared the surgical procedures during the COVID-19 shelter-in-place to those in the corresponding time periods in the years 2019 and 2018.

## Methods

We conducted a retrospective study on all ophthalmologic visits from February 17 to April 12, 2020 to capture the four weeks prior to and four weeks after scaled back scheduling began on March 16, 2020. The time period of February 17 to March 15 was considered “prior to COVID-19” because healthcare visits were not yet altered during this time period. March 16 to April 12, 2020 was considered “during COVID-19” as this was a 4-week period affected by institutional scheduling changes related to COVID-19.

We reviewed the dates of surgical case order placement and completion of surgical procedures in order to determine the urgency of procedures during this 8-week study period. We then performed a retrospective study on all ophthalmologic visits, surgical case orders, and surgical procedures for the corresponding 8-week time period in 2018 and 2019 to evaluate changes in care delivery during COVID-19 compared to the same time period in previous years, similar to the analysis of trauma patients completed by Christey et al. [3]. Surgical procedures were considered procedures performed in an operating room and were categorized using current procedural terminology (CPT) codes. Emergent procedures were defined as those that occurred within 7 days or fewer of surgical case order placement. Urgent procedures were defined as those that occurred between 8 and 21 days of surgical case order placement. We used the volume, type, and urgency of ophthalmologic surgical procedures for the corresponding time periods in 2018 and 2019 as a benchmark to which we compared the volume and type of surgeries in the 8-week study period in 2020. Billing data was used to determine visit type and diagnosis. Additionally, we compared the demographic data of patients having ophthalmologic procedures during the 8-week study periods in 2020, 2019 and 2018.

Descriptive statistics for each variable were reported. Univariate analysis used the chi-square test or Fisher’s exact test for categorical covariates. The significance level was set at P < 0.05 by two-sided test. This study was conducted as a quality improvement initiative and met criteria for determination of non-human subject research by the Emory University Institutional Review Board.

## Results

### Demographics

**Fig. 1:**
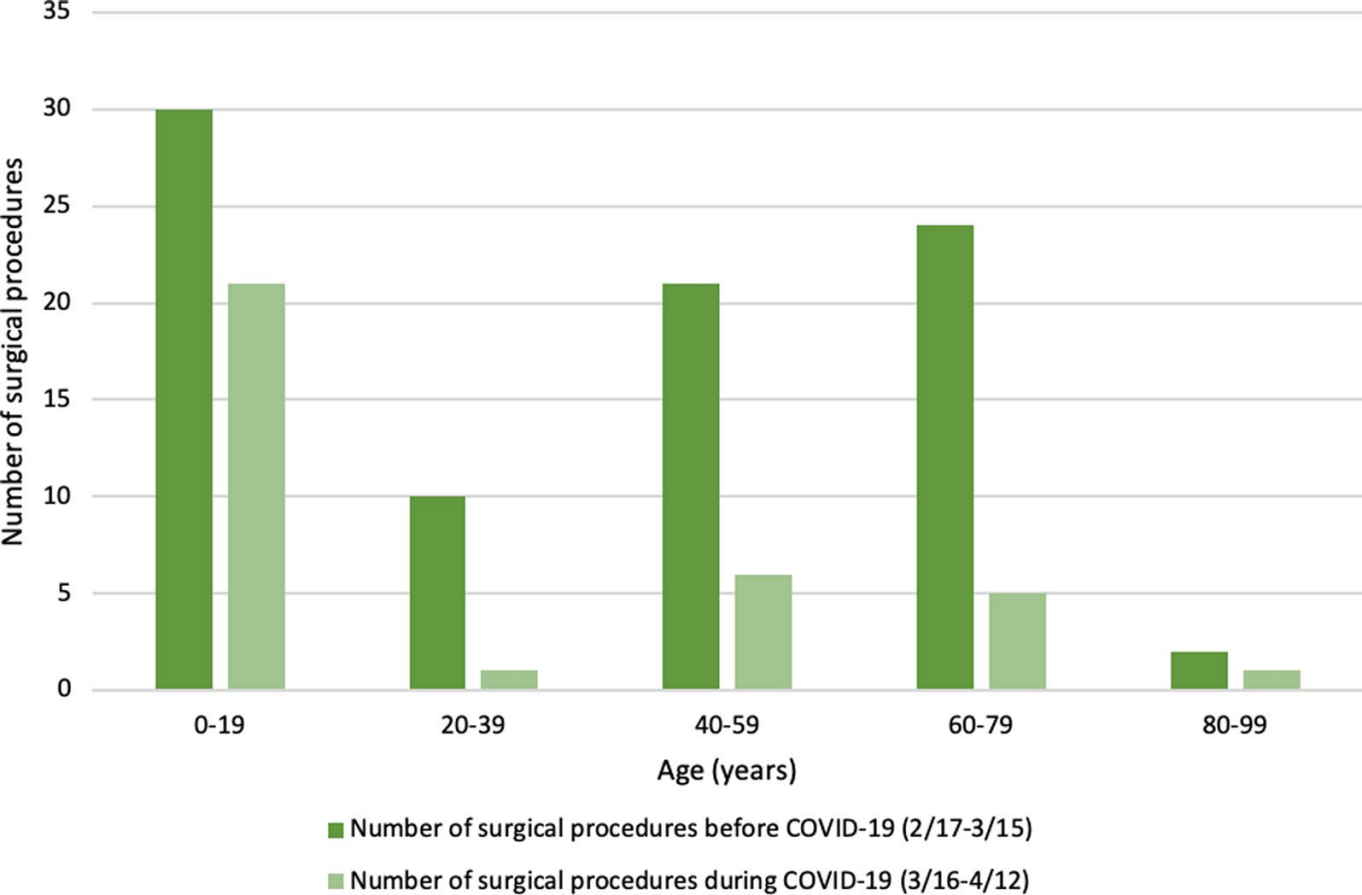
The number of surgical procedures before and during COVID-19, by age group

Demographics and other characteristics of patients who had surgical procedures between February 17 and April 12, 2020 and during the corresponding “during COVID-19” dates in 2018 and 2019 are shown in Table 1. The average age of patients having surgery before COVID-19 was 39.4 years with a range of 5 months to 95 years, while the average age for patients having surgery during the 4-week COVID-19 period was 25.7, with a range of 17 months to 91 years. Patients aged 20–39 years-old had the greatest decrease (90.0%) in surgical volume, and patients aged 0–19 years-old accounted for the majority (61.8%) of surgical procedures after March 16, 2020 (Fig. 1).

**Table 1:** Comparison of demographics for patients having surgery

Characteristics	Feb 17–March 15 2020 n=87(%)	March 16–April 12 2020 n=34(%)	p-value* (During COVID v. prior to)	March 16–April 12 2019 n=62(%)	March 16–April 12 2018 n=69(%)	p-value* (During COVID v. 2019 v. 2018)
*Gender*						
Female	33 (37.93%)	17 (50.0%)	0.2256	14 (22.58%)	30 (43.48%)	0.0098**
Male	54 (62.07%)	17 (50.0%)	–	48 (77.42%)	39 (56.52%)	–
*Age*						
Average (range)	39.4 (range: 5 months–95 years)	25.7 (range: 17 days–91 years)	–	43.5 (3 months–84 years)	42.9 (range: 7 months–80 years)	–
0–19 years old	30 (34.48%)	21 (61.76%)	0.0717	15 (24.19%)	21 (30.43%)	0.0070**
20–39 years old	10 (11.49%)	1 (2.94%)	–	11 (17.74%)	6 (8.70%)	–
40–59 years old	21 (24.14%)	6 (17.65%)	–	13 (20.97%)	13 (18.84%)	–
60–79 years old	24 (27.59%)	5 (14.71%)	–	21 (33.87%)	28 (40.58%)	–
80–99 years old	2 (2.30%)	1 (2.94%)	–	2 (3.23%)	1 (1.45%)	–
*Race*						
White or Caucasian	28 (32.18%)	7 (20.59%)	0.4586†	21 (33.87%)	25 (36.23%)	0.4975^†^
Black or African American	19 (21.84%)	2 (5.88%)	–	15 (24.19%)	20 (28.99%)	–
Other^‡^	4 (4.60%)	0 (0%)	–	0 (0 %)	1 (1.45%)	–
Unknown	36 (41.38%)	25 (73.53%)	–	26 (41.94%)	23 (33.33%)	–
*Type of insurance*						
Medicare	16 (18.39%)	2 (5.88%)	0.2730	9 (14.5%)	10 (14.49%)	<0.0001**
Medicaid	9 (10.34%)	4 (11.76%)	–	8 (12.9%)	11 (15.94%)	–
Private insurance	51 (58.62%)	22 (64.71%)	–	44 (70.97%)	43 (62.32%)	–
Not recorded	11 (12.64%)	6 (17.65%)	–	1 (1.61%)	5 (7.25%)	–

^†^White or Caucasian v. Black or African American

^‡^Other race includes Asian and Native Hawaiian or Other Pacific Islander

*The p-value is calculated by chi-square or Fisher’s exact test for categorical covariates of the 2020 data

**Statistical significance at α < 0.05

More males (n = 54 vs. 33 females) had surgery before COVID-19 while during COVID-19 the gender split was even (n = 17 vs. 17). There was a uniform decrease in the number of surgeries completed, regardless of race or insurance (Table 1). The make-up of insurance coverage for patients having surgery during COVID-19 was not significantly different from those having surgery prior to March 16, 2020. However, there was a statistically significant difference in insurance coverage for patients during COVID-19 compared to prior years (Table 1, p < 0.0001), which might be due to the increase in percentage of patients with insurance “not recorded” in their electronic medical record in 2020.

### Number of visits to the vitreoretinal surgery and uveitis division

Between February 17 and March 15, 2020, there were 2314 unique retina patient encounters, including clinic visits and surgeries (Table 2). After March 16, 2020, the absolute number of patient encounters decreased to 747 encounters (a 67.72% decline). During the corresponding time period in 2019, the division had 2231 encounters from February 17 to March 15, and 1872 encounters from March 16 to April 12 (a 16.10% decline). In 2018, there were 1796 encounters between February 17 and March 15, and 1719 encounters from March 16 to April 12 (a 4.29% decline).

**Table 2:** Number and urgency of retina surgeries

	Feb 17–March 15 2020	March 16–April 12 2020	p-value* During COVID v. prior	March 16–April 12 2019	March 16–April 12 2018	p-value* During COVID v. 2019 v. 2018
Total number of unique patient encounters	2314	747	–	1872	1719	–
Total number of surgical procedure (% of total visits)	87 (3.76%)	34 (4.55%)	0.4559	62 (3.31%)	69 (4.01%)	0.2744
Number of emergent^†^ surgical procedure (% of total surgical procedures)	23 (26.44%)	18 (52.94%)	0.0056**	21 (33.87%)	15 (21.74%)	0.0063**
Number of urgent^‡^ surgical procedure (% of total surgical procedures)	26 (28.89%)	4 (11.76%)	0.0380**	14 (22.58%)	21 (30.34%)	0.1075

^†^Emergent procedures were defined as those that occurred within 7 days or fewer of surgical case order placement

^‡^Urgent procedures were defined as those that occurred between 8 and 21 days of surgical case order placement

*The p-value is calculated by chi-square or Fisher’s exact test for categorical covariates of the 2020 data

**Statistical significance at α < 0.05

### Number, urgency, and types of surgical procedures

From February 17 to March 15, 2020, there were 87 unique surgeries completed, representing 3.76% of all retina patient encounters. Of these surgeries, 23 (26.44%) were emergent and another 26 (28.89%) were urgent (Table 2). In the 4-week time period following March 16, the number of surgeries decreased to 34, representing 4.55% of all retina patient encounters.

The absolute number of emergent cases decreased to 18 (a 21.74% decrease, p = 0.0056), whereas the percentage of all surgeries that were emergent doubled (52.94% vs. 26.44%). Emergent cases before COVID-19 included surgical procedures for retinal detachments (n = 18), vitrectomies for diabetes causing vitreous hemorrhage (n = 5), examinations under anesthesia (n = 5), and others (n = 7). During COVID-19, emergent cases were surgeries for retinal detachments (n = 15), examinations under anesthesia (n = 2), and vitrectomies for diabetes causing vitreous hemorrhage (n = 1).

**Fig. 2:**
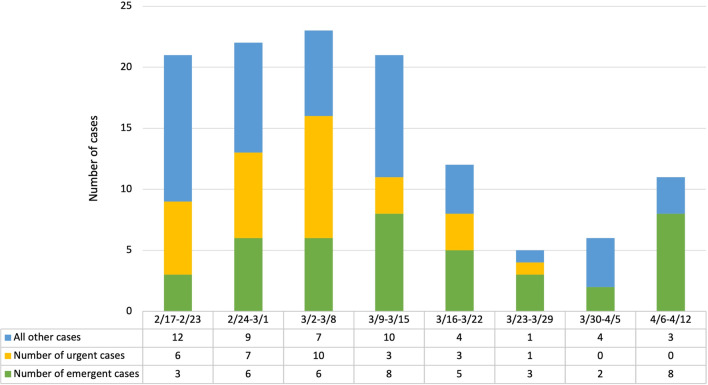
Number of emergent and urgent cases by week in 2020

The absolute number of urgent cases decreased from 26 before COVID-19 to four (an 84.62% decrease, p = 0.0380). Urgent cases before COVID-19 included procedures for retinal detachments (n = 11), macular hole surgeries (n = 8), vitrectomies for diabetes causing vitreous hemorrhage (n = 2), examination under anesthesia (n = 1), and others (n = 7). The urgent surgical procedures during COVID were two retinal detachments and one examination under anesthesia. The trend in number of emergent and urgent cases per week during the 8-week study period is illustrated in Fig. 2. One surgical procedure during the period after March 16, 2020 did not have a surgical order date and therefore the urgency/emergency of the surgery could not be evaluated.

During this same time period in 2019, from February 17 to March 15, there were 99 unique surgical procedures, representing 4.43% of all retina patient encounters. Of all surgical procedures completed, 29 (29.29%) were emergent and another 24 were urgent (24.24%). In the 4-week time period following March 16, 2019 there were 62 unique surgical procedures, representing 3.31% of all retina patient encounters. Twenty-one of these surgical procedures (33.87%) were emergent and 21 (22.58%) were urgent (Table 2).

In 2018, there were 70 unique surgical procedures completed from February 17 to March 15, representing 3.90% of all retina visits. Of all surgical procedures completed, 22 (31.43%) were emergent and 12 were urgent (17.14%). From March 16 to April 12, 2018, there were 69 unique surgical procedures, representing 4.01% of all retina visits. Fifteen (21.74%) of these surgical procedures were emergent and 21 (30.34%) were urgent (Table 2). One surgical procedure during the period before March 16, 2018 did not have a surgical order date and therefore the urgency/emergency of the procedure could not be evaluated.

Table 3 illustrates the different types of surgeries that were completed during the 8-week study period in 2020 and the time corresponding to “during COVID-19” in 2018 and 2019. The number of surgical procedures related to retinal detachments decreased from 49 to 19 in the 4 weeks before versus after March 16, 2020. In comparison, there were 28 and 27 retinal detachment surgeries during March 16-April 15, 2019 and 2018, respectively. The number of macular hole surgeries decreased from 15 to 0 in the 4 weeks before versus after March 16, 2020 (p = 0.0251). In 2019 and 2018, there were 4 and 8 macular hole surgeries, respectively, in the “during COVID-19” time period (p = 0.0471).

**Table 3:** Types of retina surgeries

	Feb 17–March 15 2020 n = 151 (%)^†^	March 16–April 12 2020 n = 44 (%)^†^	p-value* During COVID v. prior to	March 16–April 12 2019 n = 62 (%)^†^	March 16–April 12 2018 n = 69 (%)^†^	p-value* During COVID v. 2019 v. 2018
Examination under anesthesia	40 (26%)	20 (45%)	0.0165**	9 (15%)	14 (20%)	0.0007**
Retinal detachment	49 (32%)	19 (43%)	0.1887	28 (45%)	27 (39%)	0.7765
Vitrectomy for diabetes causing vitreous hemorrhage	24 (16%)	3 (7%)	0.1251	20	17	0.0007**
Macular hole surgeries	15 (9%)	0 (0%)	0.0251**	4 (6%)	8 (12%)	0.0471**
Other^‡^	23 (15%)	2 (5%)	0.0621	1 (2%)	3 (4%)	0.6594

^†^Totals may be greater than the total number of unique surgeries because all CPT codes billed for one surgery were included (e.g. examination under anesthesia in addition to another procedure)

^‡^Other procedures include: removal of extraocular implanted material, posterior segment and fluorescein angiography (includes multi-frame imaging) for a pediatric patient

*The p-value is calculated by chi-square or Fisher’s exact test for categorical covariates of the 2020 data

**Statistical significance at α < 0.05

## Discussion

Our study importantly adds to the literature investigating the impact of the COVID-19 pandemic on retina surgeries and other clinic procedures performed at a large academic institution [4–6]. We found that the absolute volume of surgeries over the 4-week period before versus after March 16, 2020 decreased from 87 to 34. This relatively large decrease is consistent with the cancellations and rescheduling in response to the pandemic. Interestingly, the 4-week period (March 17 to April 12) in 2018 and 2019 also saw a drop in the total number surgical procedures compared to the prior 4-week periods (February 15 to March 16) though to a smaller degree. This may point to a seasonal impact on the number of retina surgical procedures completed.

### Effects on the urgency of cases

The absolute number of emergent cases remained relatively consistent before versus during COVID (23 vs. 18) and across years (range: 15 to 29). As the total number of surgeries decreased after March 16, emergent cases accounted for a greater portion of all surgeries compared to before (52.94% vs. 26.44% before). In contrast, the absolute number of urgent cases decreased greatly from 26 prior to March 16 to four, which is outside the 2018–2019 range of 12 to 24 urgent cases. The decrease in urgent cases is likely to be due to recommendations to delay non-emergent cases while protocols were being created to keep patients and surgical staff safe. Alternatively, fewer patients were being evaluated in clinic after March 16, resulting in fewer diagnoses that might lead to scheduling surgery within 21 days [7]. Patients may have also foregone visits for conditions that have been lingering due to the shelter-in-place recommendations or limiting outdoor activities.

The number of emergent cases did not see the same decrease. Interestingly, the number of retinal detachment surgeries was higher (49) right before the shelter-in-place than in years prior (27 and 28), which could either be from increased referrals due to evolving operating room recommendations which may not have been easily implemented in community practices or due to a variation in the incidence of retinal detachments this year. If the latter is the case, the sudden decline during the shelter-in-place either implies that patients were not seeking care for detachments, or that limitations in activity decreased the incidence of detachments. Similar data has been published in preterm births and in care of myocardial infarctions [8–12]. This needs to be evaluated further.

Georgia’s statewide shelter-in-place was issued April 3, 2020, and, as Fig. 2 illustrates, surgical cases after this time point were more likely to be emergent and very unlikely to be urgent. For example, after April 3, there were six cases for retinal detachments, all of which were emergent. The above demonstrates the need for specialists to continue to provide specialty care not otherwise available to patients. Due to the nature of the ophthalmology examination, patients presenting with vague symptoms (blurry vision or new floaters) need to be evaluated to determine whether their case is emergent. A number of these patients will not have emergencies, but all need to be evaluated safely to determine the urgency of their underlying condition.

### Effects on age of patients having surgery

The average age of patients decreased more than 10 years, from 39.4 years to 25.7 years. Fewer older patients had surgical procedures during the 4-week COVID period while younger (pediatric) patients continued to have surgery and therefore made up more of the surgical population. Our practice and the associated children’s hospital, which was unaffected by the initial surge, sees a large number of pediatric retina patients and younger patients continued to have examinations under anesthesia and retinal detachment repairs. Younger patients seeking retina care are likely to be more emergent due to the risk of permanent vision loss from retinal detachments or the diagnosis of an intraocular tumor. Older patients may have disproportionately rescheduled or cancelled their surgeries not only because they were “elective” but also because they wanted to avoid visiting the hospital, since older age is a risk factor for serious complications related to COVID-19 [13]. With delays in these surgical procedures, the visual health of the older population may be impacted more heavily by the pandemic.

### Limitations

Limitations to this study should be noted. First, this is a retrospective study, which is subject to selection bias. In order to mitigate the effect of this potential bias, we included all patients who visited the vitreoretinal division of our academic medical center during the time periods of interest in 2018, 2019, and 2020. Second, we analyzed all patient encounters coded by physicians in the division. However, trauma surgeries completed by non-retina surgeons were not included in this study. Third, we used March 16 as the date delineating “before” and “during COVID-19” based on the healthcare institution’s recommendations, though an official stay-at-home order was not issued in Atlanta, GA until March 27 and the Georgia statewide shelter-in-place was not issued until April 3, 2020 [14, 15]. We tried to address the potential effects of this discrepancy in time points by comparing the 2020 “during COVID-19” time period to the corresponding time periods in 2019 and 2018. Additionally, we calculated the urgency of surgical cases based on the dates of surgical case order placement and the completion of surgeries. Given this data collection, we were unable to determine post-schedule alterations or the postponement of surgeries. Finally, since our academic medical center sees a large volume of pediatric patients, which may differ from other institutions, the average age of our patients decreased.

The results of our study bring to light important questions: Are patients with vision threatening illness being treated? If there are true decreases in the number of emergencies, are these related to physical activities? If patients are delaying seeking care, when do these non-urgent and non-emergent cases become urgent or emergent? What will the long-term visual outcomes on patients and on society be of delaying this care? If the volume of retinal procedures remains low throughout the pandemic, will there be a rebound effect when the pandemic subsides?

## Conclusion

In this retrospective cross-sectional study, national recommendations in response to COVID-19 impacted the number, the types and the urgency of surgical procedures completed at the vitreoretinal division of a tertiary care academic medical center as well as the demographics of patients having ophthalmologic surgical procedures. We found that older patients were affected by the rescheduling and cancellations of surgical procedures due to COVID-19, whereas pediatric patients continued to need and present for emergent surgeries. Retinal detachments continued at a similar rate, while care for urgent cases declined. These results may reveal an unmet need for older patients and those with urgent cases when national guidelines in response to a global pandemic such as COVID-19 are implemented. Future recommendations for ophthalmologists should consider these patients likely to experience reduced access to ophthalmologic care during a pandemic. The long-term effects of the national recommendations in response to COVID-19 on the visual health of the population requires further investigation.

## Data Availability

The datasets used and/or analyzed during the current study are available from the corresponding author on reasonable request.
